# Laparoscopic gastrojejunostomy to manage gastric outlet obstruction associated with endoscopic submucosal dissection of large gastric epithelial neoplasms: A two‐case report

**DOI:** 10.1002/deo2.18

**Published:** 2021-08-22

**Authors:** Takeshi Uozumi, Tetsuya Sumiyoshi, Yusuke Tomita, Kaho Tokuchi, Hiroya Sakano, Masahiro Yoshida, Ryoji Fujii, Takeyoshi Minagawa, Yutaka Okagawa, Kohtaro Morita, Kei Yane, Hideyuki Ihara, Michiaki Hirayama, Hitoshi Kondo

**Affiliations:** ^1^ Department of Gastroenterology Tonan Hospital Hokkaido Japan

**Keywords:** endoscopic submucosal dissection, gastric bypass, gastric outlet obstruction, laparoscopy, peristalsis

## Abstract

We report on two patients with stasis symptoms, including vomiting and nausea that were caused by deformity, stenosis, and decreased gastric peristalsis associated with artificial ulcers after endoscopic submucosal dissection (ESD). In both cases, the symptoms remained unresolved despite repetitive endoscopic balloon dilation (EBD). Therefore, laparoscopic gastrojejunostomy was performed. Soon after the procedure, their food intake was improved. Laparoscopic gastrojejunostomy can be an option for the treatment of gastric outlet obstruction induced by a large field of gastric ESD that is refractory to EBD.

## INTRODUCTION

Artificial ulcers associated with endoscopic submucosal dissection (ESD) of large gastric epithelial neoplasms can lead to stenosis and deformity. A wide resection area that comprises more than three‐quarters of the circumference is a significant risk factor for post‐ESD stenosis in both the cardia and antrum. Endoscopic balloon dilation (EBD) is the standard approach for post‐ESD stenosis management.^1^, ^2^, ^3^ However, the EBD perforation rate for post‐ESD stenosis is 7.8%–14.3%, indicating possible safety issues associated with EBD.^1^, ^2^ Additionally, extensive resection may lead to not only stenosis but also slow gastric emptying because of gastric deformity and decreased gastric peristalsis.^4^

Gastrojejunostomy is the standard palliative approach for gastric outlet obstruction (GOO) caused by malignancies such as gastric and pancreatic cancers. Laparoscopic gastrojejunostomy (Lap‐GJ) can achieve early recovery of bowel movement and resumption of oral feeding.^5^ This report discusses the cases of two patients who underwent Lap‐GJ to successfully resolve nausea and vomiting associated with post‐ESD GOO.

All participants provided written informed consent, and the Tonan Hospital Institutional Review Board (IRB) granted permission to review the patients’ records (IRB number: 498). The study was conducted according to the principles of the Declaration of Helsinki.

## CASE REPORTS

### Case 1

**FIGURE 1:**
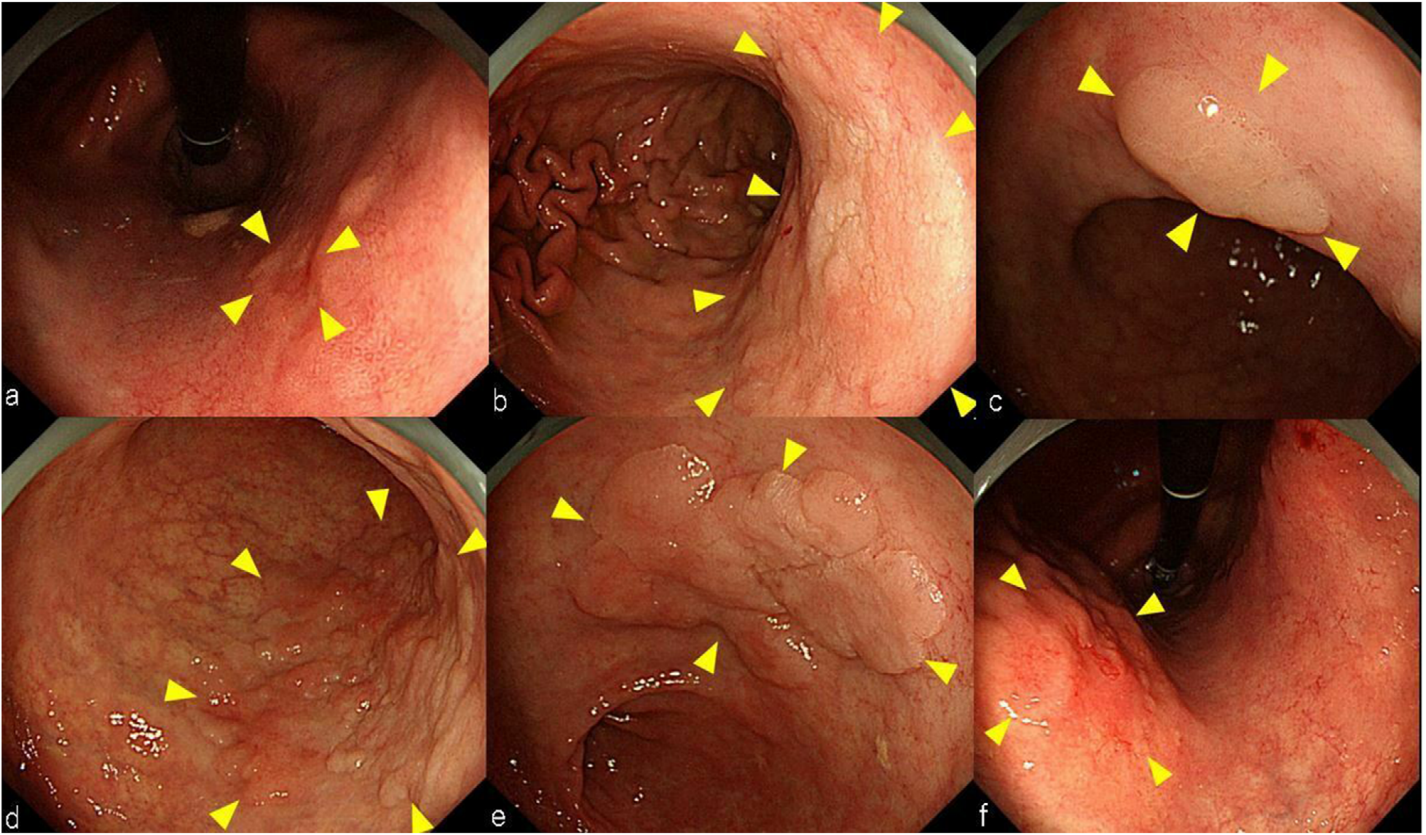
Esophagogastroduodenoscopy revealed six tumors in the gastric body and antrum of patient one (yellow triangle). (a) A 6‐mm diameter 0‐IIc lesion found on the lesser curvature of the upper body; biopsy of the resected specimen revealed possible adenocarcinoma. (b) A 40‐mm diameter 0‐IIa lesion found on the lesser curvature of the middle body; biopsy of the resected specimen revealed possible adenocarcinoma. (c) A 10‐mm diameter 0‐IIa lesion found on the lesser curvature of the angle; biopsy of the resected specimen revealed adenoma. (d) A 50‐mm diameter 0‐IIa lesion extending from the posterior wall to the greater curvature of the angle; biopsy of the resected specimen revealed possible adenocarcinoma. (e) A 25‐mm 0‐IIa lesion found on the lesser curvature of the antrum; biopsy of the resected specimen revealed adenoma. (f) A 25‐mm 0‐IIa lesion found on the anterior wall of the middle body; biopsy of the resected specimen revealed possible adenocarcinoma

**FIGURE 2:**
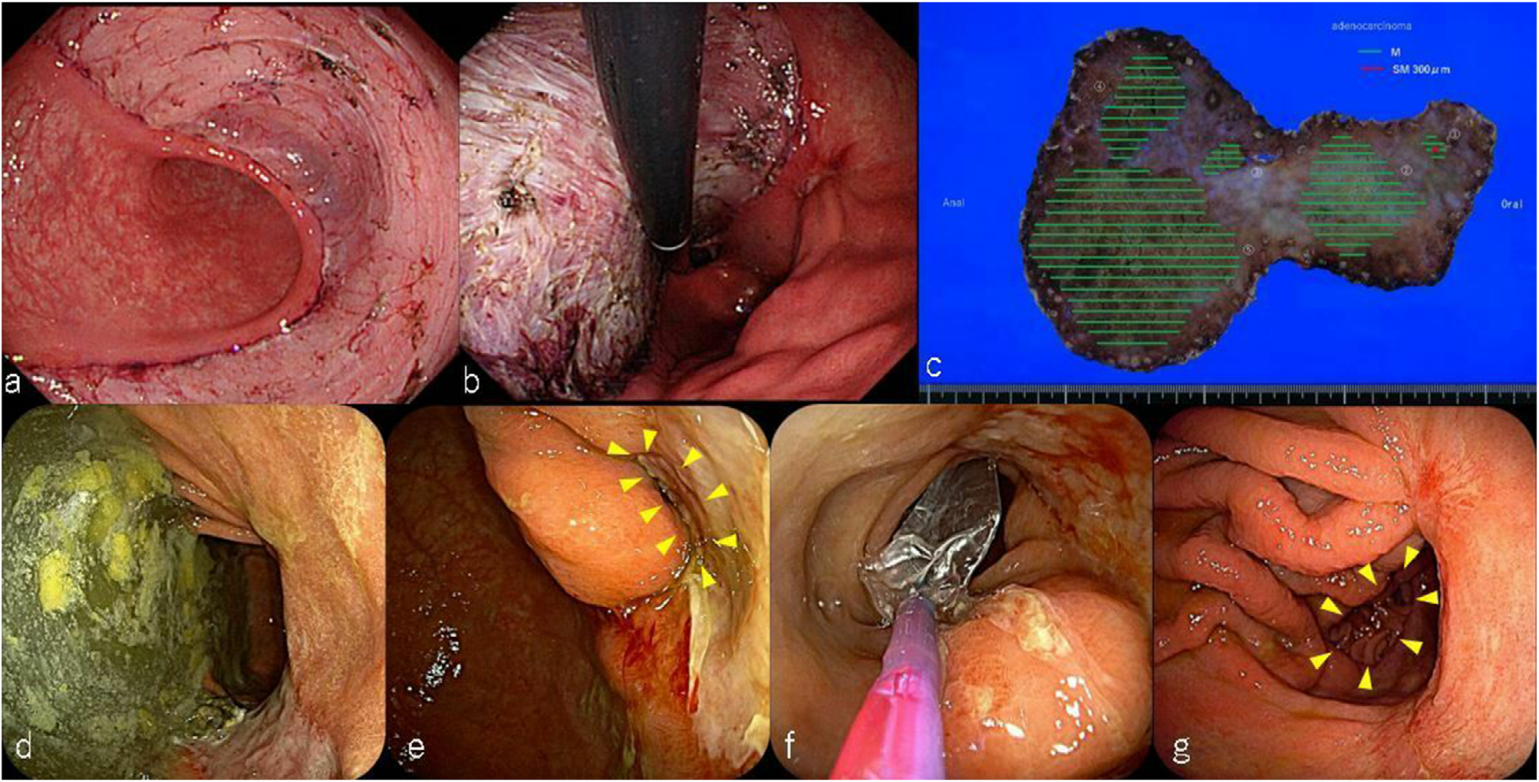
(a) The mucosal defect in the antrum spanned three‐quarters of the circumference. (b) The mucosal defect caused by endoscopic submucosal dissection extended to the lesser curvature of the upper body. (c) The resected specimen measured 175 × 125 mm, and the resection margins were tumor‐negative. (d) A large quantity of residual food was found inside the stomach. (e) Gastric deformity and stenosis, extending from the antrum to the lower body, were observed; the lumen is marked by the yellow triangle. (f) Endoscopic balloon dilation was performed, and the luminal diameter was expanded to 20 mm. (g) Endoscopic images after laparoscopic gastrojejunostomy

An 83‐year‐old male underwent esophagogastroduodenoscopy (EGD) as part of an investigation for epigastric pain, which revealed six tumors in the gastric body and antrum. Since the six tumors were spread across a large field (Figure 1a–f), total gastrectomy was recommended by our surgeon. However, the patient strongly wanted to preserve his stomach, and therefore, we carefully described the risk of post‐ESD GOO and performed ESD. A 25‐mm diameter 0‐IIa lesion located in the anterior wall of the middle body was removed first because it was suspected to have invaded the submucosal layer (Figure 1f). A histopathological examination of the resected specimen revealed a well‐differentiated adenocarcinoma with a diameter of 28 mm in the mucosal layer. Given the low risk of lymph node metastasis, another ESD was performed for the remaining lesions 49 days after the first ESD. Resecting all the tumors separately appeared to be difficult as their margins were close to one another; therefore, they were resected en bloc. The procedure time for ESD was 293 min. The mucosal defect, which extended from the gastric upper body to the antrum, spanned three‐quarters of the circumference in the antrum (Figure 2a and b). Histopathologically, the resected specimen was 175 × 125 mm in diameter (Figure 2c). Based on the Japanese gastric cancer treatment guidelines, curative resection was achieved for all lesions.^6^ One month later, the patient developed gastric stasis symptoms, including nausea, vomiting, abdominal distention, and loss of appetite. EGD indicated deformity and stenosis extending from the upper body to the antrum in the stomach in addition to residual food in the stomach (Figure 2d and e). The endoscope (EG‐L590WR; Fujifilm) barely passed beyond the deformed area, but the stasis symptoms persisted. The luminal diameter was only approximately 10 mm; therefore, to relieve the symptoms, EBD was performed four times during 1 month, which consequently expanded the diameter to 20 mm (Figure 2f). However, the patient's symptoms persisted, and his weight decreased from 50 to 42 kg. Hence, Lap‐GJ with Billroth Ⅱ plus Braun anastomosis was performed with a procedure time of 133 min. Soon after the surgery, the patient could ingest food without developing nausea or vomiting; however, he needed rehabilitation because of muscle weakness. The patient was hospitalized for 16 days after Lap‐GJ. His weight recovered gradually, reaching 50.5 kg 1 year postoperatively. The follow‐up EGD, which was performed 1 year after the surgery, revealed that there was no residual food in the stomach (Figure 2g).

### Case 2

**FIGURE 3:**
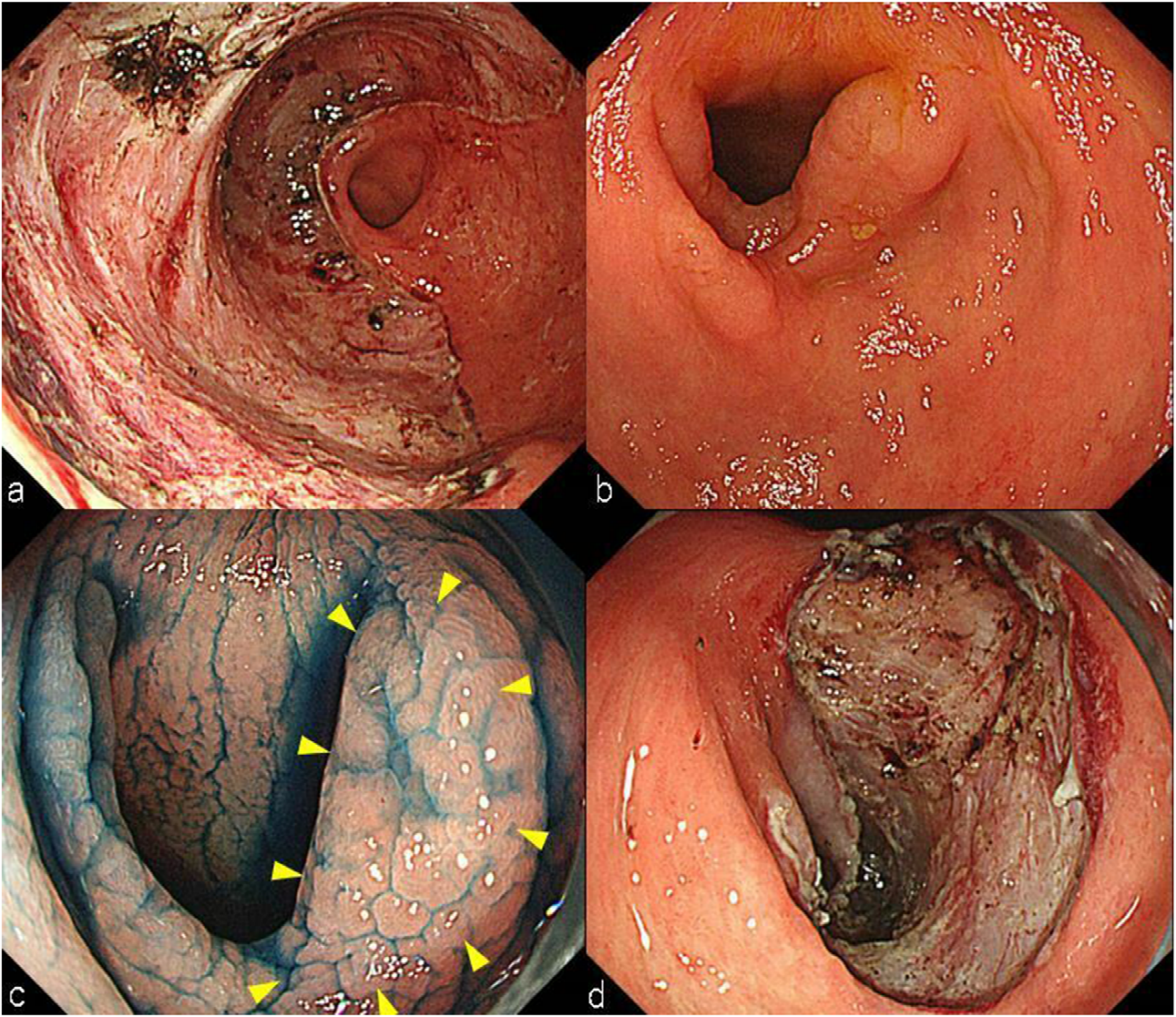
(a) The mucosal defect in the antrum caused by previous endoscopic submucosal dissection (ESD) spanning three‐quarters of the circumference. (b) Deformity in the antrum, caused by the previous ESD, was observed. (c) An ill‐defined 0‐IIa lesion was found in the antrum deformed by the previous ESD scar (yellow triangle). (d) The mucosal defect in the antrum reached the size of half of the circumference

**FIGURE 4:**
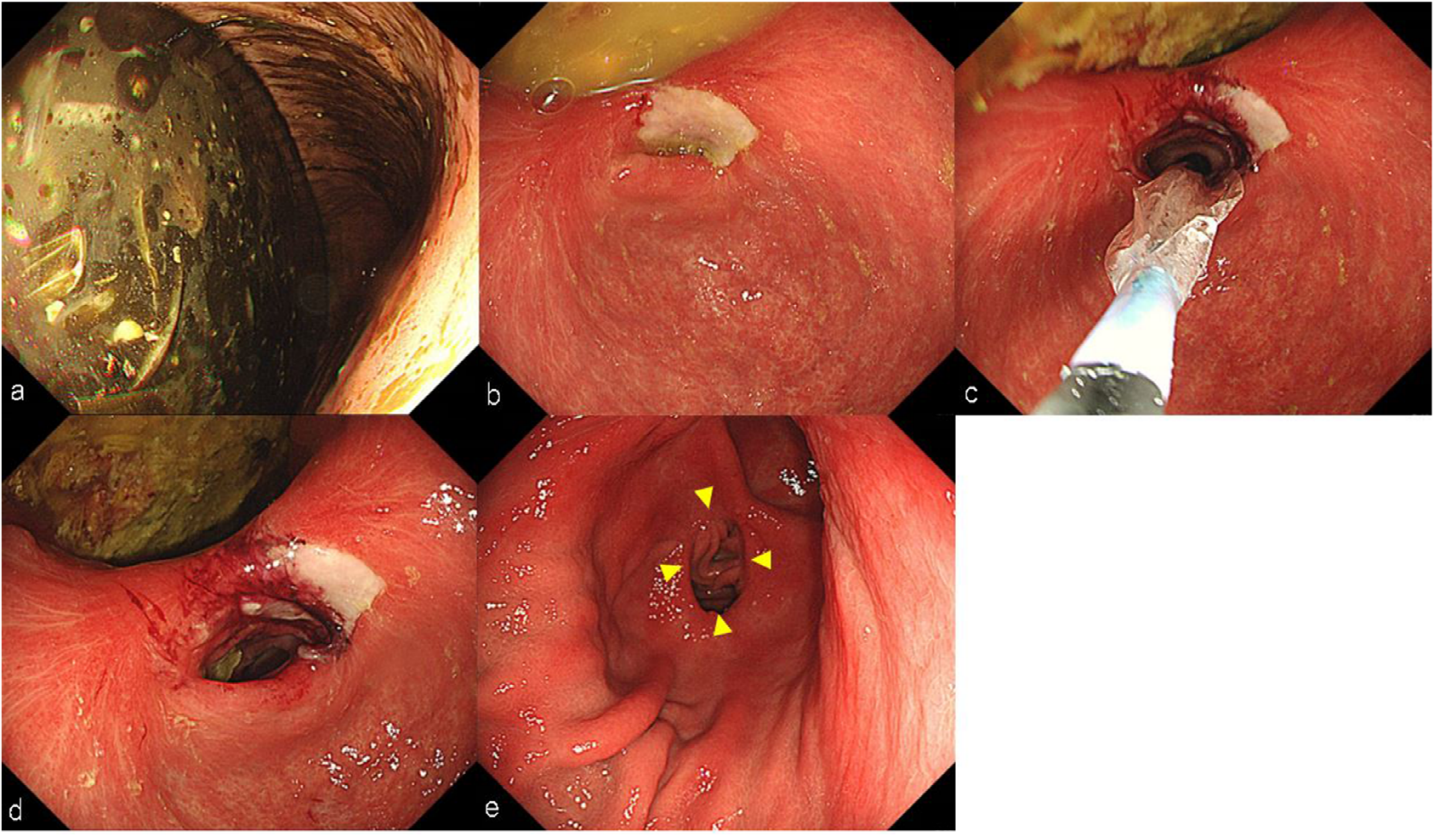
(a) A large quantity of residual food was found inside the stomach. (b) Deformity and stenosis of the antrum were observed. (c) Endoscopic balloon dilation was performed. (d) The luminal diameter was expanded to 15 mm. (e) Endoscopic images after laparoscopic gastrojejunostomy

An 83‐year‐old female had previously undergone ESD for gastric cancer that extended from the lesser curvature to the anterior wall of the antrum; curative resection had been achieved. The large field of artificial ulcer, which comprised three‐quarters of the antrum, resulted in a post‐ESD deformity (Figure 3a and b). The patient underwent a follow‐up EGD after ESD, and a 10‐mm diameter 0‐IIa lesion with unclear lateral margin was identified in the posterior wall of the antrum (Figure 3c). ESD was performed for the lesion with a procedure time of 50 min. A histopathological examination revealed that the resected specimen was a well‐differentiated adenocarcinoma confined to the mucosal layer. The mucosal defect comprised half the circumference of the antrum (Figure 3d). One month later, the patient experienced nausea and vomiting. EGD revealed deformity and stenosis encompassing the antrum in addition to residual food in the stomach (Figure 4a and b). The endoscope (GIF‐HQ290; OLYMPUS) barely passed beyond the deformed area, but the stasis symptoms persisted; hence, to relieve the symptoms, the patient underwent EBD eight times during 80 days (Figure 4c and d). However, the patient's symptoms persisted, and she remained unable to ingest food. The patient wanted the symptoms to be resolved immediately; therefore, Lap‐GJ with Billroth Ⅱ plus Braun anastomosis was performed having a procedure time of 173 min. Soon after the surgery, the patient could ingest food without developing nausea or vomiting. No complications were associated with Lap‐GJ; however, the patient needed rehabilitation because of muscle weakness. She was hospitalized for 19 days. Her weight did not change, but follow‐up EGD performed 1 year after the surgery revealed no residual food in her stomach (Figure 4e).

## DISCUSSION

ESD is generally applied for treating gastric epithelial neoplasms with low metastatic risk, regardless of their size.⁶ However, wide resection of the gastric epithelium in the antrum or cardia can result in stenosis and deformity, and ESD that reaches three‐quarters of the circumference is reportedly a significant risk factor for developing post‐ESD stenosis.^1^, ^2^, ^3^ The reported rate of developing stenosis after gastric ESD is 0.8%–2.5%.^7^ EBD, which is the standard therapeutic approach for post‐ESD stenosis, typically requires repetition. Patients generally receive between four and nine EBD procedures for post‐ESD stenosis, with a median of 50 treatment days.^1^, ^2^ The rate of perforation, which is a common EBD complication, is 1.5% per procedure.^3^ A study with a small sample size found that the perforation rate of EBD for stenosis after gastric ESD was 7.8%–14.3%.^1^, ^2^ Although EBD can alleviate stasis symptoms, it is associated with a risk of perforation.

Local steroid injection into artificial ulcers caused by ESD might prevent gastric deformities and stenosis in patients with gastric epithelial neoplasms; however, these findings were only based on small cohorts.^4^ Local steroid injections for artificial ulcers following gastric ESD do not show sufficient evidence for preventing stenosis and deformity. In addition, the risk of cytomegalovirus‐associated gastric ulcer was reported in a previous case report, which might have been caused by immunosuppression associated with local steroid injection.^8^ Therefore, steroid injection was not performed in our cases.

Peristaltic dysfunction is also reported as a cause of GOO. Peristaltic dysfunction is speculated to be caused by injury of Latarjet's branch of the vagal nerve during ESD in the lesser curvature of the upper stomach.^9^ In Case 1, the large field of ESD extending to the lesser curvature of the upper stomach might have caused peristaltic dysfunction and led to stasis symptoms.

Although EBD is effective in treating post‐ESD stenosis, stasis symptoms caused by gastric deformity and decreased gastric peristalsis may be intractable. There are some reports about noninvasive GOO avoidance methods such as polaprezinc administration, but there is insufficient evidence to support its use. Therefore, surgical intervention is necessary for GOO that does not improve even after repetitive EBD. Elderly patients are easily enfeebled by poor food intake; therefore, shortening the stasis symptom period is essential for them. In addition, frequent vomiting related to intractable GOO will cause aspiration pneumonia, which may increase mortality in elderly patients. Lap‐GJ can achieve early recovery of bowel movement and resumption of oral feeding.⁵ Lap‐GJ is less invasive than distal gastrectomy, and successfully ameliorated stasis symptoms in our cases. Lap‐GJ may promote early discharge and a stable life at home, leading to good clinical outcomes for elderly patients. A drawback of Lap‐GJ is that deformity and stenosis of the preserved stomach remains. It may lead to poor examination and missed diagnosis of metachronous gastric cancer, which sometimes occurs after ESD over a long‐term period; hence, Lap‐GJ may not be recommended for younger patients.

In conclusion, Lap‐GJ can be an option for the treatment of post‐ESD GOO refractory of EBD, especially in elderly patients.

## CONFLICT OF INTEREST

The authors declare that there is no conflict of interest.

## FUNDING INFORMATION

None.
